# Catalpol Regulates Oligodendrocyte Regeneration and Remyelination by Activating the GEF-Cdc42/Rac1 Signaling Pathway in EAE Mice

**DOI:** 10.1155/2022/7074157

**Published:** 2022-11-25

**Authors:** Minghui Wu, Qi Kang, Yuezhi Kang, Yanping Tong, Tao Yang, Yongping Fan

**Affiliations:** Beijing Tiantan Hospital, Capital Medical University, Beijing, China

## Abstract

The main obstacle to remyelination in demyelinating diseases, such as multiple sclerosis, is the inability of oligodendrocyte precursor cells (OPCs) to differentiate into mature oligodendrocytes (OLs) in the demyelinating region. Consequently, promoting OL differentiation and myelin remodeling is a key goal in the search for treatments. Rho GTPases play diverse and important roles throughout the development of neuronal axons and the formation of the myelin sheath. The current study aimed to investigate the direct protective effects of catalpol on demyelination damage induced by myelin oligodendrocyte glycoprotein (MOG) immunization and to explore whether the GEF-Cdc42/Rac1 signaling pathway contributes to the regeneration effect induced by catalpol. In the MOG-induced experimental autoimmune encephalomyelitis (EAE) mouse model of demyelination, we observed that catalpol significantly promoted OL development by enhancing the expression of glutathione S-transferase pi (GST-pi) in the affected brain. By Luxol fast blue staining and myelin basic protein (MBP) expression assessment, catalpol was found to increase MBP expression and promote myelin repair. Furthermore, catalpol promoted OL differentiation associated with the upregulation of Cdc42/Rac1 expression and activation in vivo. In addition, PAK1/MRCK*α*, proteins downstream of Cdc42/Rac1, was positively regulated by catalpol. We also found that catalpol alleviated clinical neurological dysfunction, inhibited inflammatory infiltration, increased the proportion of Treg cells, and suppressed demyelination. Overall, our study is the first to reveal that catalpol can promote OL generation and myelination and contributes to the crucial regulatory process of GEF-Cdc42/Rac1 signaling expression and activation. Therefore, catalpol is a promising drug candidate for the potential treatment of demyelinating diseases.

## 1. Introduction

Oligodendrocytes (OLs) and myelin are key structures for maintaining the integrity of axonal structures and the transmission of nerve signals by providing trophic and metabolic support [1]. Myelin sheath destruction leads to disruption of axonal conduction and severe neurological dysfunction [2]. In the central nervous system (CNS), myelin formation is caused by the migration of OL precursor cells (OPCs) to white matter sites rich in axons, followed by differentiation into mature OLs, and then by the extension of complex protrusions around the axons [3]. The promotion of OLs regeneration may help to restore CNS function and to prevent further axonal degeneration. However, the process of OL maturation and regeneration is impeded by multiple factors under pathological conditions, such as multiple sclerosis (MS), which leads to failure of spontaneous myelin regeneration and thus results in long-term, progressive disease [4, 5]. As a result, the discovery of therapeutic drugs that would promote the ability of OPCs to differentiate and regenerate myelin in demyelinated areas would be a significant step forward in MS treatment [6].

The Rho-family small GTPases are membrane-anchored intracellular signal transducers that bind and hydrolyze GTP; these enzymes can transduce extracellular signals to intracellular signals to regulate cell survival, proliferation, and differentiation. The Rho-family GTPase pathway permits cells to perform a variety of cellular activities, including cell cycle control, membrane trafficking, and actin cytoskeleton rearrangement, all of which have significant impact on cell motility, deformation, and fate [7–9]. The role of Rho GTPases in signaling has been extensively studied because many clinical disorders have been linked to Rho GTPase malfunction or dysregulation, making them excellent potential targets for drug treatment [10–12]. They act as key intracellular molecular switches by alternating between their active GTP-bound form and inactive GDP-bound form, and their activation requires efficient nucleotide substitution of GTP for GDP through the catalytic action of guanine nucleotide exchange factors (GEFs) [13, 14]. OPCs differentiation toward mature myelinating OLs is characterized by the expression of myelin-associated genes and by morphological changes in the cells. Transcriptome analysis has been performed to confirm that Cdc42 and Rac1 are expressed by OPCs and OLs in the CNS and regulated by their development [15]. As OLs develop, they sense extracellular signaling stimuli and regulate their intrinsic signaling pathways as well as the organization of their actin cytoskeleton [16]. The expression and activity of Cdc42 and Rac1 increase as OL differentiation proceeds; these proteins are able to bind to effector proteins in active situations and thus participate in various signaling pathways [17]. Several lines of research have demonstrated crucial roles for Cdc42 and Rac1 in the morphological differentiation of OLs and in myelin formation activity [18, 19]. In the current study, we tested whether catalpol promotes neural functional recovery and myelin regeneration in mice with experimental autoimmune encephalitis (EAE). We established an EAE model control group and compared it with drug-treated EAE groups in terms of functional scores, histopathological observation, microstructural observations, and protein and gene expression mainly to observe the beneficial effects of catalpol on myelin regeneration and neuroinflammation. We speculate that catalpol may activate GEFs in OPCs through integrin receptors or transmembrane proteins on the cell membrane, and the activated GEFs may then convert GTP to GDP and transfer a phosphate to Cdc42/Rac1 to activate it [20]. As a result, increasing phosphorylated Cdc42/Rac1 reactivates downstream proteins, allowing OPCs to switch from migration to the radial membrane extension necessary for myelination, thus positively affecting OPCs differentiation toward OLs. There are few studies on this signaling pathway in OL differentiation, and we believe that it may be an important novel regulatory remyelination mechanism for demyelinating illnesses.

Catalpol, a major active compound in the Chinese herb *Rehmannia*, has good oral availability [21, 22]. We previously reported that catalpol improves neurological impairment and remyelination in EAE mice [23, 24], but the mechanism remains unclear. Herein, we further investigated the underlying mechanism by which catalpol promoted axonal remyelination in an EAE model induced by myelin oligodendrocyte glycoprotein (MOG) immunization. Cdc42/Rac1 acts as an intracellular signaling transducer that can be modulated by a variety of extracellular inputs. The study of the GEF-Cdc42/Rac1 signaling pathway provides new ideas for the use of catalpol to treat demyelinating diseases and promote myelin regeneration.

Our experimental study showed that catalpol exerted powerful nerve regeneration effects in EAE mice. We demonstrated that catalpol can efficiently promote the differentiation and maturation of OLs and accelerate remyelination. We further explored the mechanisms by which drugs regulate OL maturation. We interpret our findings to indicate that catalpol can positively facilitate OL regeneration by promoting the activation of the GEF-Cdc42/Rac1 signaling pathway, suggesting that this drug is a promising candidate for the clinical treatment of demyelinating diseases.

## 2. Materials and Methods

### 2.1. EAE Induction and Catalpol Treatment

Eighty female C57BL/6 mice of specific-pathogen-free (SPF) grade were bred under SPF conditions and used at 6–8 weeks of age. All experiments and protocols were performed under the approval of the Institutional Animal Care and adhered to the guidelines of the Animal Welfare Committee at Capital Medical University (AEEI-2019-186). The mice were randomized into four different groups (*n* = 20, each group): (1) normal control (NC), (2) EAE + phosphate-buffered saline (PBS), (3) EAE + 0.3 mg/kg fingolimod (FTY720, Novartis, Basel, Switzerland), and (4) EAE + 40 mg/kg catalpol [24] (CA, Shanghai Yuanye Bio-Technology Co., Ltd., Shanghai, China).

The EAE model was induced by subcutaneous immunization with 200 *μ*L of emulsion containing 50 *μ*g of MOG peptide 35–55 (MOG_35–55_) (Beijing Scientific Biotechnology Co., Ltd., Beijing, China) and 300 *μ*g of *Mycobacterium tuberculosis* (Sigma–Aldrich, St. Louis, MO, United States) in a 1 : 1 mixture with complete Freund's adjuvant (CFA) (Sigma-Aldrich) at four different sites on the back. All immunized mice were intraperitoneally (i.p.) injected with 200 ng of pertussis toxin (PTX, Sigma-Aldrich) in 100 *μ*L of PBS on days 0 and 2 postimmunization (p.i.). NC mice were vaccinated with the same emulsion, only without MOG, and they were not given pertussis toxin. Catalpol was administered by daily intragastric gavage for 40 days for prophylactic treatment. Additionally, catalpol at 40 mg/kg was the optimal dosage to combat EAE mice according to our previous study [24, 25]. FTY720 was administered by gavage as a positive control drug after the disease onset, and PBS was administered before symptoms appeared. The NC and PBS treatment groups received the same volume of PBS once a day for 40 days.

### 2.2. Body Weight and Clinical Behavioral Assessments

After the EAE model was established, the body weight progression and clinical disability scores of the animals were assessed daily for 40 days in a blind manner. The clinical signs of EAE were used to assign disease scores according to the following 0–5 scale [26]: 0, no clinical symptoms; 0.5, stiff tail; 1, paralyzed tail; 1.5, paralyzed tail and waddling gait; 2, paralysis of one hind limb; 2.5, paralysis of one hind limb and weakness of another limb; 3, complete paralysis of both hind limbs; 3.5, paralysis of the hind limbs and weakness of the forelimbs; 4, quadriparesis/moribund state; and 5, death.

### 2.3. Histopathological Staining Analysis

Mice were anesthetized and sacrificed in the acute (day 20 p.i.) and remission (day 40 p.i.) phases and transcardially perfused with PBS and 4% paraformaldehyde (PFA). For pathological assessment, brain samples were carefully harvested, placed in 4% PFA for 24 h, and then embedded in paraffin for sectioning. Neural tissues were cut into 5 *μ*m sections and subjected to hematoxylin and eosin (H&E) staining for inflammatory infiltration and Luxol fast blue (LFB) for demyelination. Quantitative analysis of inflammatory infiltration and demyelination levels was performed as previously described [27]. The degree of inflammatory infiltration was evaluated on a scale from 0 to 4, as follows: 0, no inflammatory cells; 1, sporadic inflammatory cells; 2, some inflammatory cells and karyopyknosis; 3, inflammatory cell infiltration surrounding blood vessels and perivascular cuff formation; and 4, extensive inflammatory cell infiltration and karyopyknosis or diffuse infiltration of inflammatory cells [28]. The percentage of area myelinated was measured as the ratio of the blue area to the total area of the corpus callosum. ImageJ software (NIH, Bethesda, USA) was used to count the number of infiltrating cells in inflammatory foci per section and measure the percentage of demyelinated area.

### 2.4. Flow Cytometry Analysis

Briefly, cells (1 × 10^6^) were resuspended in 100 *μ*L of flow cytometry staining buffer and stained with appropriate conjugated antibodies or isotype-matched control antibodies on ice.

Cells were isolated from fresh spleens and passed through a 100 *μ*m cell strainer (Becton-Dickinson) to prepare a single-cell suspension and then used to optimize staining conditions. Cells were resuspended in 100 *μ*L FACS buffer before cell surface staining with antibodies against relevant surface markers, FITC-conjugated anti-CD4 (diluted 1 : 100, 35-0042, Tonbobio), and APC-conjugated anti-CD25 (diluted 1 : 100, 20-0251, Tonbobio). Cells were then washed, fixed, and permeabilized with Transcription Factor Fixation/Permeabilization buffer (TNB-1020-KIT, Tonbobio) for 20 min at 4°C according to the manufacturer's protocol. Then, Foxp3 staining was performed with PE-Cy7-conjugated anti-Foxp3 (diluted 1.5 : 100, 60-5773, Tonbobio). Data were acquired on a FACSAria (Becton–Dickinson LSRFortessa, San Jose, CA) and analyzed using FlowJo software (Treestar, Ashland, OR).

### 2.5. Transmission Electron Microscopy (TEM) of Brain Lesions

For ultrastructural analysis of myelin, the brain tissue was fixed in 4% PFA overnight; next, the corpus callosum was dissected out and perfused with 2.5% glutaraldehyde and flushed with 0.1 M phosphate buffer (PB, pH 7.4) three times. Then, the specimen was postfixed in 1% osmium acid, dehydrated with alcohol, and embedded in a pure embedding agent. Serial sections (70 nm) were cut with a diamond knife and mounted on formvar-coated mesh copper grids. Random electron micrographs (∼90 micrographs per group) were taken of the ultrathin slices by TEM (H-7700, Hitachi, Japan). G-ratios [29] were calculated by dividing the measured inner axonal diameter by the total outer axonal diameter in ImageJ, using at least 100–200 randomly selected myelin axons.

### 2.6. Immunofluorescence Staining

For immunofluorescence analysis, paraffin-embedded brains were deparaffinized and rehydrated. After being permeabilized with 0.3% Triton X-100 for 30 min and blocked with 1% bovine serum albumin (BSA) for 1 h, brain sections were incubated with primary antibodies against MBP (1 : 200, 10458-1-AP, Proteintech), GST-pi (1 : 500, 312, MBL), NG2 (1 : 200, ab12905, Abcam), GEF (1 : 500, ab201687, Abcam), Rac1 (1 : 20, ARC03, Cytoskelet), and Cdc42 (1 : 500, ab187643, Abcam) overnight at 4°C. The corresponding secondary antibodies, conjugated with ALexa Fluor 488 or ALexa Fluor 594 (1 : 400, Abcam), were added, and the samples were incubated for 1 h at room temperature (RT). Nuclei were counterstained with DAPI. Brain images were collected by fluorescence microscopy. Data are presented in standardized form as the mean ± SD and reported as the number or percentage of positive elements.

### 2.7. Gene Expression Analyses

For quantitative real-time PCR (qRT-PCR) analysis, the total RNAs were extracted from the brains of mice with a HiPure Total RNA Mini Kit (Magen, Guangzhou, China), and the RNA was reverse transcribed into cDNA with a HiScript III-RT SuperMix kit (Vazyme, Nanjing, China) according to the manufacturer's instructions. Next, first-strand cDNA samples were assayed using the Fast SYBR Green method (Vazyme, Nanjing, China) on a CFX Connect Real-Time PCR Detection System (Bio–Rad, Hercules, USA) and were analyzed using CFX management software v2.0. Experiments were performed in triplicate, and each target gene's relative mRNA expression level was determined according to the 2^−ΔΔCt^ method, with GAPDH as the internal control [30]. The normalized expression is presented as the fold induction over the control sample. The primer sequences were as follows:

GAPDH, forward primer 5′-CCTCGTCCCGTAGACAAAATG-3′ and reverse primer 5′-TGAGGTCAATGAAGGGGTCGT-3′; GEF, forward primer 5′-TCTTCACCACCATCTCCGTCTC-3′ and reverse primer 5′-CTTTACAGCGGTTGTGGATGGT-3′; Cdc42, forward primer 5′- TGCCAAGAACAAACAGAAGCC-3′ and reverse primer 5′-CAGCACTTCCTTTTGGGTTGAG-3′; Rac1, forward primer 5′-CCGATTGCCGACGTGTTCT-3′ and reverse primer 5′-GTGGTGTCGCACTTCAGGATAC-3′; MBP, forward primer 5′-GGACAGTGATGTGTTTGGGGAG-3′ and reverse primer 5′-TCTCGGGAAAAGAGGCGGA-3′;GST-pi, forward primer 5′-GATGGAGACCTCACCCTTTACC-3′ and reverse primer 5′-GATGGAGACCTCACCCTTTACC-3′; and Olig2, forward primer 5′-AGCAATGGGAGCATTTGAAG-3′ and reverse primer 5′- CAGGAAGTTCCAGGGATGAA-3′.

### 2.8. Western Blot Analysis

Tissue samples were lysed in RIPA lysis buffer containing a protease inhibitor and a phosphatase inhibitor (Applygen Technologies Inc., Beijing, China). The protein concentrations of all samples were measured using the BCA Protein Assay Kit (Applygen Technologies Inc.). Protein samples (equal amounts per lane) were run on Criterion SDS–PAGE gels (Bio–Rad) and transferred onto polyvinylidene difluoride (PVDF) membranes (Millipore, Darmstadt, Germany). The transfer membranes were blocked in blocking buffer (TBS with 0.1% Tween 20 (TBST) and 5% skim milk) for 1 h at RT followed by incubation with the following primary antibodies: anti-NG2 (1 : 1000, ab129051, Abcam), anti-Olig2 (1 : 500, MABN50, Millipore), anti-GST-pi (1 : 800, 312, MBL), anti-MBP (1 : 1000, GB12226, Servicebio), anti-GEF (1 : 1000, ab201687, Abcam), anti-Rac1 (1 : 500, ARC03, Cytoskelet), anti-phospho-Rac1 (1 : 1000, 2641, CST), anti-Cdc42 (1 : 1000, 2466S, CST), anti-MRCK*α* (1 : 1000, 81681S, CST), anti-PAK1 (1 : 1000, 2602, CST), anti-Tubulin (1 : 20,000, 66031-1, Proteintech), anti-GAPDH (1 : 20,000, 60004-1, Proteintech), and the corresponding HRP-conjugated anti-IgG antibodies. Finally, the protein bands were detected using a gel chemiluminescence imaging system and quantified by determining the grayscale values using ImageJ. All experiments were repeated three to four times.

### 2.9. Statistical Analysis

All statistics were performed using GraphPad Prism 8.0 software (GraphPad, San Diego, USA). Datasets were examined for normal distribution. Data are presented as the mean ± standard deviation (SD) from at least three independent experiments. Parametric analyses comparing multiple groups were performed by one-way analysis of variance (ANOVA) followed by Tukey's post hoc test. Values of *P* <0.05 were considered to be significant.

## 3. Results

### 3.1. Catalpol Attenuates Clinical Progression and Neurological Disability in EAE Mice

**Figure 1:**
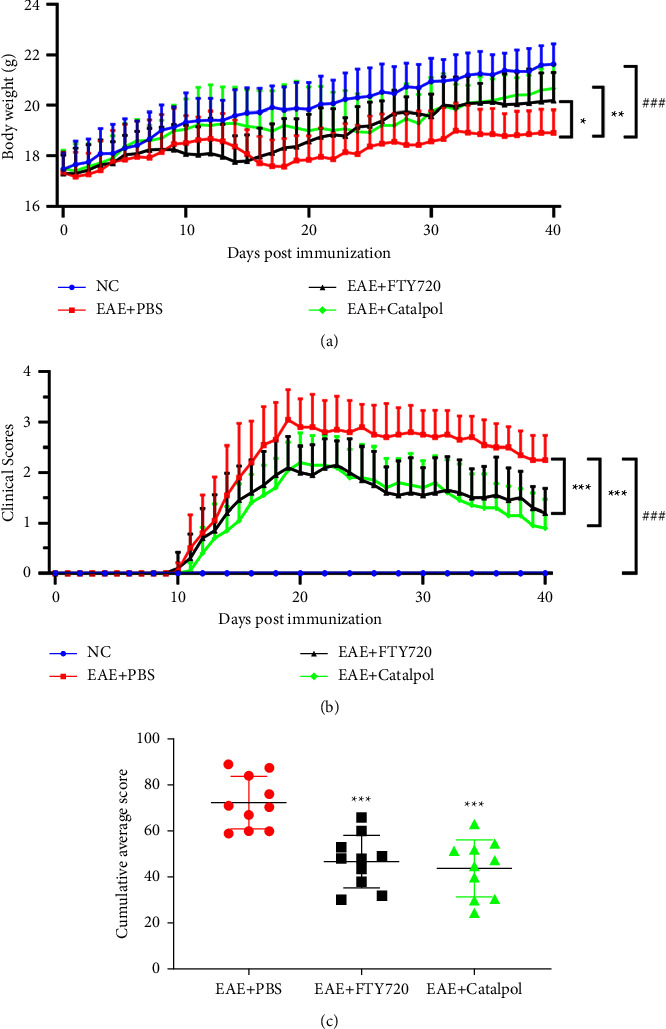
Catalpol attenuates clinical progression and neurological disability in EAE mice. C57BL/6 EAE mice were immunized with MOG_35–55_ and treated with PBS, FTY720, or catalpol. (a) Comparison of body weight changes in EAE mice among groups. (b) Comparison of clinical scores of EAE mice among groups. Catalpol significantly ameliorated the clinical severity of EAE. (c) The cumulative clinical scores among groups. Data are presented as the mean ± SD analyzed by one-way ANOVA with Tukey's post hoc multiple comparison test. ^###^*P* < 0.001 versus NC group; ^∗^*P* < 0.05, ^∗∗^*P* < 0.01, and ^∗∗∗^*P* < 0.001 versus EAE + PBS group.

The prophylactic administration of catalpol demonstrated a therapeutic effect in the early stages of the disease. The body weight and daily average score data revealed that catalpol and FTY720 had significant therapeutic effects. The body weights of PBS-treated mice noticeably decreased compared with those of NC mice from days 13 to 40 p.i., and the catalpol and FTY720 treatment approach alleviated this situation (Figure 1(a)). The onset of clinical signs in PBS-treated mice occurred at day 10 p.i., and the clinical neurological scores peaked at day 20-21 p.i. Compared to the rapid increase in the PBS group, the catalpol and FTY720 groups had considerably reduced average clinical scores from days 15 to 40 p.i. (*P* < 0.001, Figure 1(b)). In conclusion, Figure 1 convincingly demonstrates that catalpol treatment observably suppresses illness severity and improves clinical disease with time (Figure 1(c)).

### 3.2. Catalpol Treatment Effectively Ameliorates CNS Immunomodulation and Neuroinflammation

**Figure 2:**
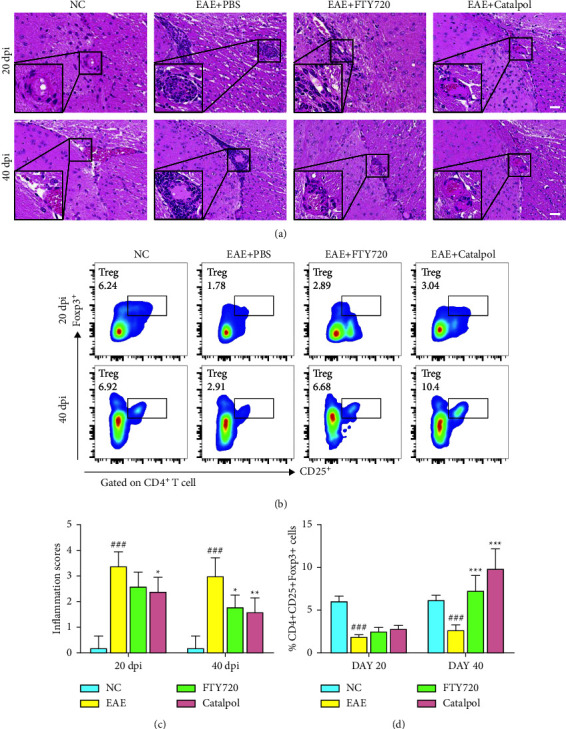
Catalpol alleviates inflammatory infiltration and ameliorates CNS immunomodulation in EAE mice. (a) Sections were observed for inflammatory infiltration by H&E staining at 20 dpi and 40 dpi. Scale bars: 100 *μ*m. (b) Percentages of CD4^+^CD25^+^Foxp3^+^ Treg cells were determined in representative flow cytometry dot plots at 20 dpi and 40 dpi; gated on CD4^+^ cells (gray box, Foxp3^+^ Treg cells). The numbers above the dot plots represent the frequency of Treg cells. (c) Quantification analysis of H&E-stained scores. (d) Quantification of the percentages of Foxp3^+^ Treg cells. Data are presented as the mean ± SD analyzed by one-way ANOVA with Tukey's post hoc multiple comparison test. ^###^*P* < 0.001 versus NC group; ^∗^*P* < 0.05, ^∗∗^*P* < 0.01, and ^∗∗∗^*P* < 0.001 versus EAE + PBS group.

Multiple sclerosis is characterized by autoimmunity and by demyelination of the neurons and degeneration of the axons in the CNS as a result of chronic inflammation [31]. We wanted to explore whether catalpol could target immunomodulation and neuroinflammation in an EAE model. In a parallel experiment, brain tissues from five mice in each group were taken and sectioned at 20 dpi, which was contrasted with 40 dpi. Histopathological images showed extensive lymphocytic infiltration and typical perivascular cuff formation in the brains of PBS-treated mice. Catalpol and FTY720 treatment dramatically alleviated inflammatory infiltration, as observed by H&E staining (*P* < 0.05, as shown in Figures 2(a)–2(c)). In addition, we investigated the effect of catalpol on neuroinflammation-associated Treg cell activation. Flow cytometric data demonstrated a significantly higher proportion of CD4^+^CD25^+^Foxp3^+^ Treg cells with catalpol and FTY720 treatment in contrast to PBS treatment at 40 dpi (*P* < 0.001, as shown in Figures 2(b)–2(d)). Taken together, the results indicated that catalpol inhibited the infiltration of inflammatory cells and ameliorated immunomodulation in the CNS of EAE mice.

### 3.3. Catalpol Treatment Leads to Enhanced OL Accumulation and Remyelination

**Figure 3:**
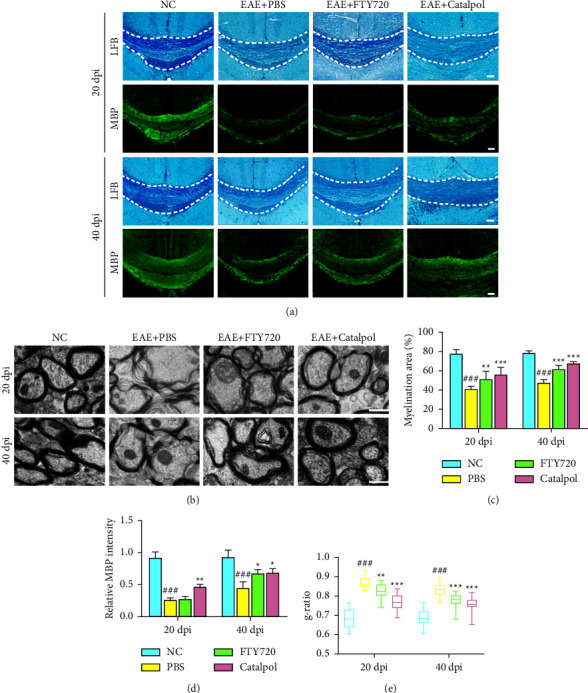
Effect of catalpol treatment in preventing demyelination and promoting remyelination in the EAE demyelinating model. (a) Sections were subjected to evaluation of myelinated areas by LFB staining in the corpus callosum and MBP expression by fluorescence microscopy at 20 dpi and 40 dpi. Scale bars: 100 *μ*m. (b) Representative myelin ultrastructure of brain tissues was observed by electron micrographs at 20 dpi and 40 dpi. Scale bars: 1 *μ*m. (c) Quantification analysis of myelinated areas. (d) Quantification analysis of MBP intensity. (e) Quantitative comparison of the g-ratio (axon diameter/fiber diameter) of myelinated fibers. Data are presented as the mean ± SD analyzed by one-way ANOVA with Tukey's post hoc multiple comparison test. ^###^*P* < 0.001 versus NC group; ^∗^*P* < 0.05, ^∗∗^*P* < 0.01, and ^∗∗∗^*P* < 0.001 versus EAE + PBS group.

Another pathological feature of MS is the formation of focal demyelinating lesions. Consequently, the demyelination level of the EAE model was evaluated by LFB staining and MBP immunostaining in the corpus callosum [32]. As shown in Figures 3(a)–3(c), the catalpol-treated group had smaller demyelinated areas and higher MBP intensity than the PBS-treated group. The expression of MBP, a myelin-specific marker, was assessed by immunostaining. At 20 dpi, severe demyelination (MBP^−^) was observed in the brains of PBS-treated mice, as some areas lost MBP staining. At 40 dpi, spontaneous recovery from demyelination in PBS-treated mice was modest, whereas remyelination in catalpol-treated mice was greatly improved (Figures 3(a)–3(d)). Taken together, our data showed that catalpol could reduce demyelination and significantly improve remyelination.

Furthermore, we evaluated myelin based on myelin ultrastructure under TEM. We observed that normal axons of the brain tissue were elliptical with uniform morphology, the structure of the myelin layer was clearly visible, and the fibers were densely arranged, with no vacuoles present. In comparison, axonal swelling, myelin thinning, and laxity were observed in the brains of untreated EAE mice. Apparent destruction of the myelin sheath structure was found in EAE mice, which was prominently prevented, and myelin thickness was increased by catalpol and FTY720 treatment (Figure 3(b)). The degree of demyelination and remyelination was quantitatively analyzed using the g-ratio (Figure 3(e)). Catalpol therapy prominently reduced the g-ratio, reflecting its protective effect on axon and myelin sheath integrity in EAE mice.

**Figure 4:**
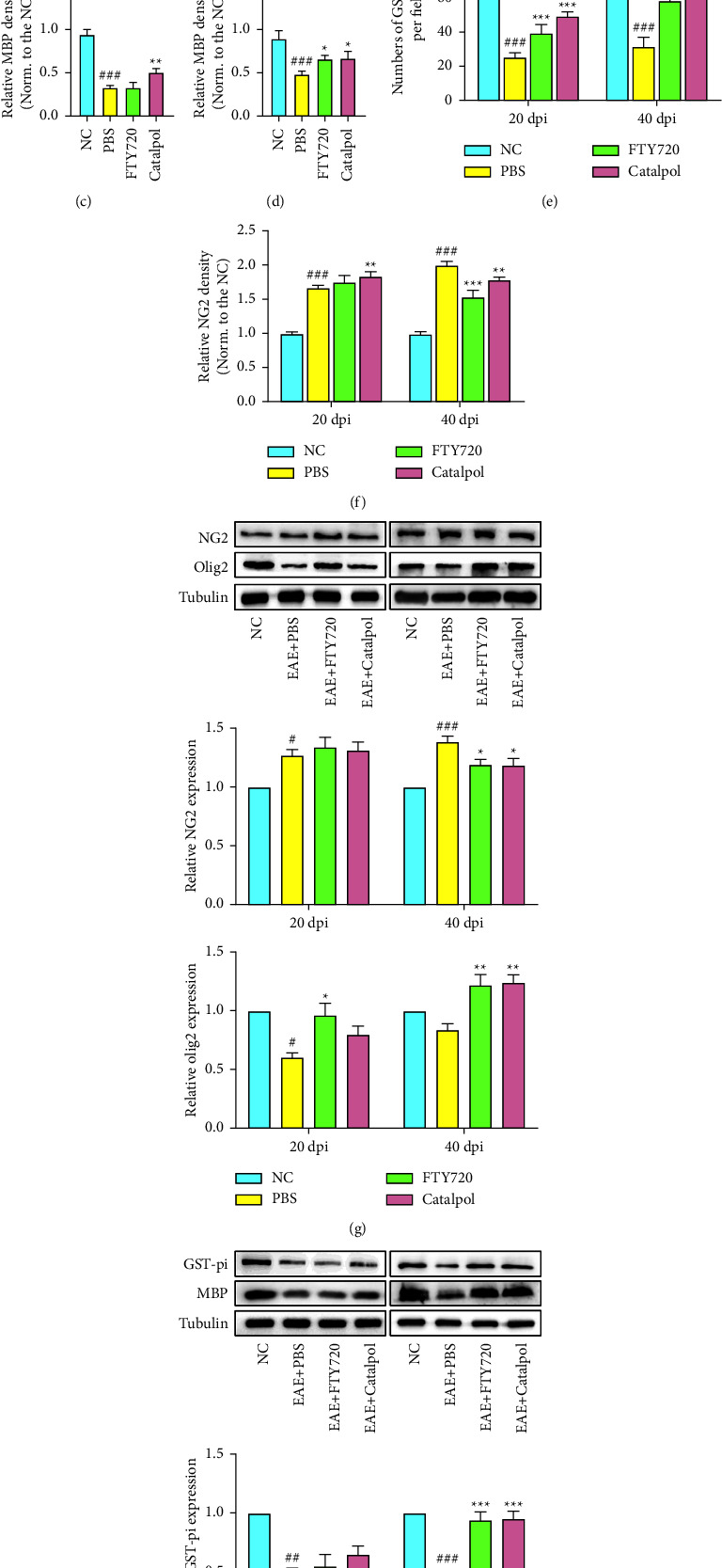
Effects of catalpol on myelin protein expression in OLs in EAE mice. Representative immunofluorescence images in the corpus callosum showing MBP (a), GST-pi (b), and NG2 (b) at 20 dpi and 40 dpi in EAE mice. Scale bar: 100 *μ*m in (a); 50 *μ*m in (b). Quantitative analysis of MBP expression (c) (d), GST-pi expression (e), and NG2 expression (f) among groups. Representative western blotting images show the expression of NG2 and Olig2 (g) and GST-pi and MBP (h) at 20 dpi and 40 dpi. Quantitative analysis of relative NG2, Olig2, GST-pi, and MBP levels is done. Protein expression was standardized using Tubulin as a sample loading control, and quantification is presented in each panel. (i) The mRNA expression of Olig2, GST-pi, and MBP at 20 dpi was measured by qRT-PCR and normalized to that of GAPDH. (j) The mRNA expression of Olig2, GST-pi, and MBP at 40 dpi was detected by qRT-PCR and normalized to that of GAPDH. Data are presented as the mean ± SD analyzed by one-way ANOVA with Tukey's post hoc multiple comparison test. ^#^*P* < 0.05, ^##^*P* < 0.01, and ^###^*P* < 0.001 versus NC group; ^∗^*P* < 0.05, ^∗∗^*P* < 0.01, and ^∗∗∗^*P* < 0.001 versus EAE + PBS group.

Additionally, to further verify that the enhancement of myelin regeneration by catalpol is due to the increased conversion from OPCs to mature OLs, we evaluated the numbers of mature OLs and OPCs by immunofluorescence and western blotting with antibodies against glutathione S-transferase (GST)-pi and MBP (markers of mature OL) and NG2 (a marker of OPC) in the same region, respectively. The expression of NG2 was found to be significantly higher in the EAE model group at 20 dpi than in the NC group (*P* < 0.001, Figures 4(b) and 4(f)), with no significant differences between the catalpol, FTY720, and PBS groups. This suggested that myelin loss stimulates the proliferation of OPCs. Compared to PBS treatment, catalpol treatment for 40 dpi led to a significantly greater number of MBP^+^ and GST-pi^+^ OL-lineage cells while a reduced of NG2^+^ OPCs (*P* < 0.05, as shown in Figures 4(a)–4(e)). These findings provide evidence that the substantial drop in new OLs in EAE mice is not attributable to OPC proliferation abnormalities, considering that catalpol may accelerate the differentiation process from OPCs to mature OLs. Consistent with the immunofluorescence data, western blot analysis showed that catalpol and FTY720 treatment substantially upregulated the expression of mature OL markers (GST-pi and MBP) and OL transcription factors (Olig2) (*P* < 0.05, as shown in Figures 4(g) and 4(h)). Furthermore, we analyzed the mRNA levels of Olig2, GST-pi, and MBP and found that catalpol treatment upregulated their expression (Figures 4(i) and 4(j)). The small disparity in the mRNA data and protein (western blotting) data could be explained by the fact that mRNA expression does not always predict protein expression levels. In general, the analysis results indicated the beneficial effects of catalpol in myelin repair and probably enhanced the differentiation of endogenous OPCs into remyelinating OLs.

### 3.4. Catalpol Regulates the Expression of the GEF Pathway to Promote OL Generation

**Figure 5:**
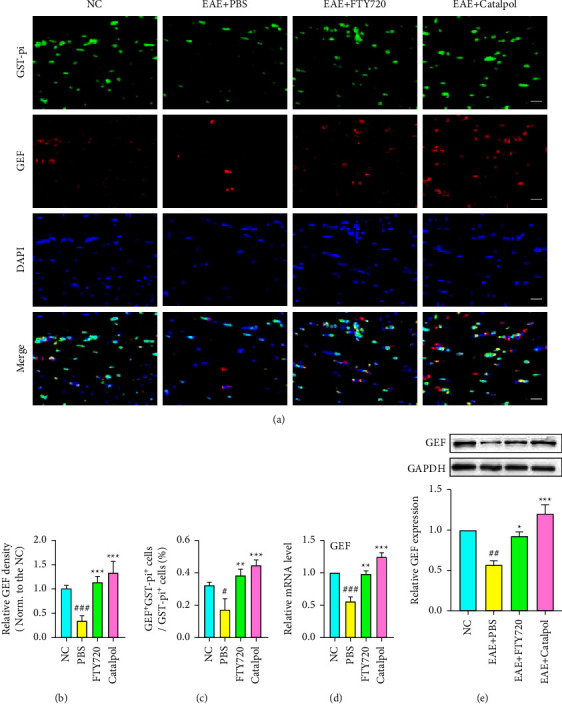
The therapeutic effect of catalpol on the upregulation of the GEF pathway and the maturation of OLs in EAE mice. (a) Representative double immunostaining images of GST-pi (green) and GEF (red) showed significantly increased numbers at 40 dpi. Scale bar: 20 *μ*m. (b) Quantitative analysis of GEF density. (c) Quantitative comparison was performed for the proportion of GEF^+^GST-pi^+^ cells among groups. (d) The mRNA expression of GEF was measured by qRT-PCR and normalized to that of GAPDH. (e) Representative western blot images display the expression of GEF at 40 dpi and quantitative analysis of the relative GEF level. Data are shown as the mean ± SD analyzed by one-way ANOVA with Tukey's post hoc multiple comparison test. ^#^*P* < 0.05, ^##^*P* < 0.01, and ^###^*P* < 0.001 versus NC group; ^∗^*P* < 0.05, ^∗∗^*P* < 0.01, and ^∗∗∗^*P* < 0.001 versus EAE + PBS group.

Activation of Rho-family proteins requires the exchange of bound GDP for GTP, which is mediated by GEF. Considering the role of GEF in cell morphology development and activation of Cdc42 and Rac1, we aimed to test the possibility that the increase in GEF activity promotes OL development in vivo and modulation of GEF by catalpol. We started by comparing their fluorescent expression in the CNS at 40 dpi by the colocalization of GST-pi^+^ and GEF^+^ and revealed that almost no GEF staining was found in PBS-treated mice, and the number of OLs (GEF^+^GST-pi^+^) was also reduced significantly (Figure 5(a)). However, GEF expression was found to be increased in EAE mice treated with catalpol and FTY720 (*P* < 0.001, as shown in Figure 5(b)), and mice treated with catalpol had a significantly greater number of OLs (GEF^+^GST-pi^+^) (*P* < 0.001, Figure 5(c)). We further investigated the expression of GEF in each group by western blot and qRT-PCR analyses (*P* < 0.05, Figures 5(d) and 5(e)). These assays showed lower expression of GEF in the PBS-treated EAE mice than in the NC group. However, the level of GEF was elevated significantly with catalpol and FTY720 treatment. Along with the immunofluorescence data, the results provide evidence that catalpol upregulates GEF expression and is favorable for OL development or remyelination in EAE mice.

### 3.5. Catalpol Enhanced OL Development by Activating the Cdc42/Rac1 Signaling Pathway in EAE Mice

**Figure 6:**
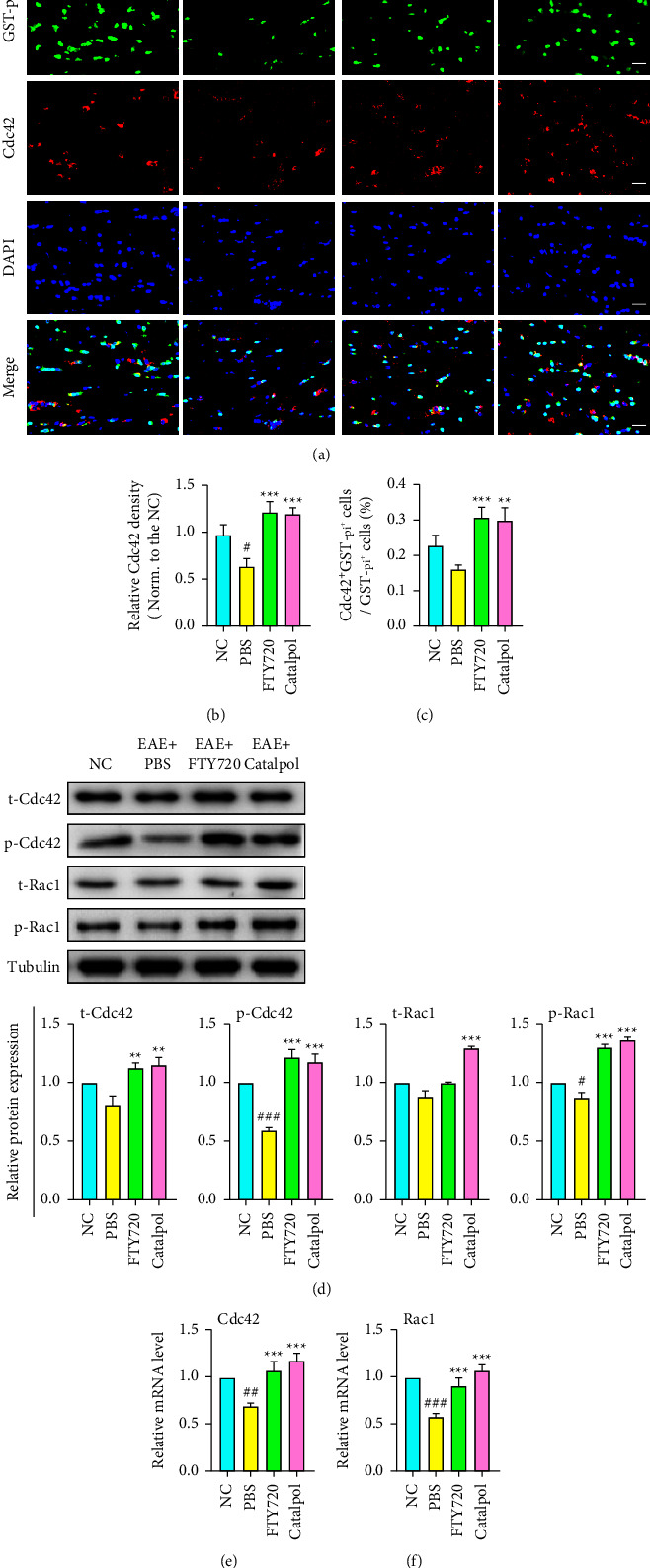
Catalpol treatment activates Cdc42 to enhance OL development in EAE mice. (a) Representative double immunostaining images of GST-pi (green) and Cdc42 (red) at 40 dpi. Scale bar: 20 *μ*m. (b) Quantitative analysis of Cdc42 density among groups. (c) Quantitative analysis was performed to determine the proportion of GEF^+^ GST-pi^+^ cells. (d) Representative western blot images display the expression of total Cdc42/Rac1 and phospho-Cdc42/Rac1 at 40 dpi and quantitative analysis of relative total Cdc42/Rac1 and phospho-Cdc42/Rac1 levels. (e, f) The mRNA expression levels of Cdc42 and Rac1 were detected by qRT-PCR and normalized versus GAPDH. Data are shown as the mean ± SD analyzed by one-way ANOVA with Tukey's post hoc multiple comparison test. ^#^*P* < 0.05, ^##^*P* < 0.01, and ^###^*P* < 0.001 versus NC group; ^∗∗^*P* < 0.01 and ^∗∗∗^*P* < 0.001 versus EAE + PBS group.

**Figure 7:**
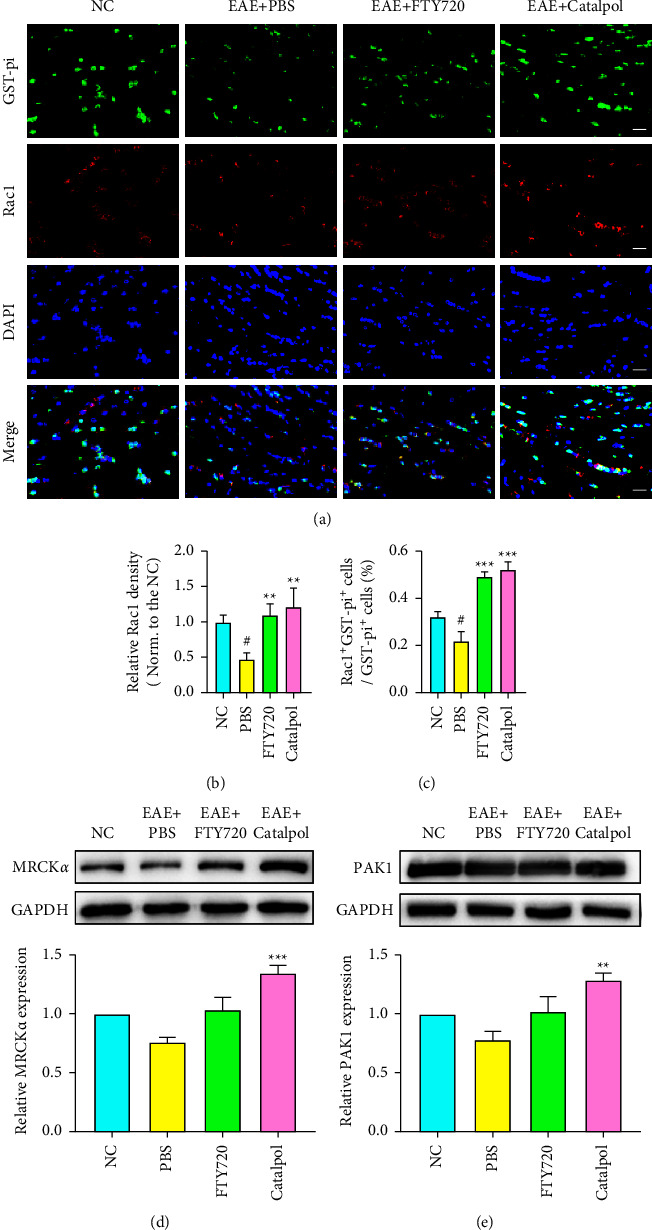
Catalpol treatment upregulates Rac1 expression to enhance OL development in EAE mice. (a) Representative double immunostaining images of GST-pi (green) and Rac1 (red) at 40 dpi. Scale bar: 20 *μ*m. (b) Quantitative analysis of Rac1 density. (c) Quantitative analysis was performed for the proportion of Rac1^+^GST-pi^+^ cells. (d) Representative western blot images show the expression of MRCK*α* at 40 dpi and quantitative analysis of the relative MRCK*α* level. (e) Representative western blotting images show the expression of PAK1 at 40 dpi and quantitative analysis of the relative PAK1 level. Data are shown as the mean ± SD analyzed by one-way ANOVA with Tukey's post hoc multiple comparison test. ^#^*P* < 0.05 versus NC group; ^∗∗^*P* < 0.01 and ^∗∗∗^*P* < 0.001 versus EAE + PBS group.

The Rho-family GTPases Cdc42 and Rac1 can regulate actin cytoskeleton remodeling, which is essential for cell motility. Previous research has illustrated that the Cdc42/Rac1 pathways play important roles during OL-lineage progression [19, 33, 34]. Thus, we hypothesized that catalpol might control OL differentiation by regulating Cdc42/Rac1. We then set out to clarify whether catalpol can stimulate the expression of Cdc42/Rac1 and their downstream target proteins while promoting OL differentiation. We first observed their expression in the CNS by coimmunostaining of Cdc42, Rac1, and the mature OL marker GST-pi with fluorescent probes in each group (Figures 6(a) and 7(a)); we observed that the expression of Cdc42 and Rac1 was significantly reduced in PBS-treated mice at 40 dpi compared with the NC group, while the percentages of OLs (Cdc42^+^GST-pi^+^ and Rac1^+^GST-pi^+^) were decreased (*P* < 0.05, Figures 6(b) and 7(b)). More Cdc42^+^GST-pi^+^ OLs and Rac1^+^GST-pi^+^ OLs accumulated in the lesions after catalpol and FTY720 treatment than after PBS treatment (*P* < 0.01 or *P* < 0.001, Figures 6(c) and 7(c)).

Next, we investigated Cdc42/Rac1 activation at the biochemical level. We further measured the mRNA levels and protein expression levels at 40 dpi. Western blot analysis showed that EAE significantly decreased the levels of phospho-Cdc42/Rac1 and total Cdc42/Rac1 expression (Figure 6(d)). However, both the total amount and the phosphorylation of Cdc42 and Rac1 were rescued by catalpol and FTY720. Additionally, the mRNA levels showed consistent results (Figures 6(e) and 6(f)). Furthermore, in this experiment, we assessed the expression of several downstream proteins related to the Cdc42/Rac1 signaling pathway: Myotonic dystrophy kinase-related Cdc42-binding kinase isoform *α* (MRCK*α*) and p21 protein-activated kinase 1 (PAK1). Western blot results showed a prominent elevation of MRCK*α* and PAK1 expressing in catalpol- and FTY720-treated mice compared with PBS-treated mice (*P* < 0.01 or *P* < 0.001, Figures 7(d) and 7(e)). Cdc42/Rac1 activation by catalpol and FTY720 leads to downstream MRCK*α*/PAK1 protein expression. Overall, in agreement with the immunofluorescence data, these observation results demonstrated that catalpol effectively contributes to endogenous Cdc42 and Rac1 expression and activation to enhance OL development.

## 4. Discussion

MS is an autoimmune CNS inflammatory demyelinating disease caused by immune cell infiltration that results in focal demyelinating lesions [35]. The EAE model is ideal for studying pathological changes in MS. Some herbal-derived ingredients with anti-inflammatory and neuroprotective properties and few side effects that may help to improve the patient condition and recovery from EAE are gaining popularity. We demonstrate in the present study that catalpol improved neurological function symptoms and alleviated neuroinflammation and damage to myelin in the brains of EAE mice, suggesting a significant neuroprotective and regenerative role. First, in a MOG-induced EAE mouse model of demyelination, we performed clinical neurological function scores and body weight monitoring for 40 days. The results showed that catalpol treatment decreased disease severity and promoted neurological recovery in EAE mice at the onset and peak and improved functional outcomes at later stages of the disease. Moreover, the results of H&E and flow cytometric analysis indicated that the catalpol treatment approach suppresses proinflammatory cell infiltration and increases the number of Treg cells. Foxp3^+^ Treg cells are a subpopulation of T cells with immunomodulatory functions in the body and a strong immunosuppressive capacity [36]. In general, Tregs are known to suppress the production of proinflammatory cytokines. Considering the modulatory effect of catalpol treatment on Tregs in EAE, catalpol may exert anti-inflammatory and immunomodulatory effects by increasing the proportion of peripheral Treg cells. Additionally, LFB staining and TEM results showed reduced demyelinated areas and thickened myelin with catalpol treatment. In conclusion, catalpol possesses good anti-inflammatory, remyelinating and neuroprotective effects. It would be worthwhile to further explore the therapeutic potential of this compound, including the mechanisms of its therapeutic action in demyelinating diseases.

Myelination is an essential process for the proper transmission of nerve impulses. The existence of various demyelinating diseases, in which myelin deficiencies produce changes in nerve impulse transmission, emphasizes the necessity of myelination [37, 38]. In demyelinating diseases, such as multiple sclerosis, the failure of myelin regeneration is mainly due to the cessation of the maturation process despite the accumulation of OPCs at the foci during remission [39]. Consequently, promoting the maturation of OPCs into myelinating OLs is crucial for myelin repair. At present, there are no effective pharmacological treatments to enhance myelin regeneration and neuroprotection in clinical applications. In the current study, the therapeutic potential of catalpol as a promyelinating and neurorestorative therapy is clearly emphasized by several pieces of evidence.

To further validate the role of catalpol in facilitating the maturation process of OLs to promote myelin regeneration, we scored the neurological function of mice after the onset of the disease and combined these data with the expression data from tissue immunofluorescence. OLs express different transcription factors and proteins at different times. The neuroglial antigen NG2, a cell surface glycoprotein expressed during the early differentiation phase of OPCs, is the main source of nascent OLs production at demyelinated foci [40, 41]. GST-pi is a cytosolic isoenzyme used as a specific marker protein for mature OLs in the mammalian brain [42, 43]. MBP is expressed only in mature OL, and one of its principal functions is to organize the compaction of OL plasma membranes to form myelin [44]. When myelin is lost, OPCs in the brain are rapidly activated, and proteoglycan NG2 expression is increased. In the acute phase, a substantial number of NG2^+^ OPCs were observed in the brains of MOG-induced EAE mice of all groups. In the remission phase, we found that markedly increased MBP^+^ and GST-pi^+^ expression in the catalpol group was not accompanied by an increase in NG2^+^ compared to the level observed in untreated EAE mice; this was considered due to stunted OPC differentiation. These observations indicate that treatment with catalpol significantly promoted the differentiation of OL, and myelin repair was considered to have occurred.

Rho-family GTPases are signaling proteins that link cellular stimuli regulating actin cytoskeleton organization, cell survival, and cell cycle progression [8]. Among these, Cdc42 and Rac1 synergistically regulate myelination and play a key role in regulating the process of compact myelin formation in oligodendrocytes. Some previous studies revealed that GTPases expressed in OL cultures resulted in the conclusion that Cdc42 and Rac1 are positive regulators that induce cytoskeleton rearrangement and promote morphological differentiation [18]. As illustrated by the morphological transformations that occur during differentiation, myelin formation is also a process of cytoskeletal rearrangement, regulated by the Rho GTPase family [45]. Multiple lines of evidence have identified crosstalk between Rho-family GTPases to promote myelin regeneration [46–48]. Activation of Cdc42 and Rac1 by Dock3 overexpression exerted neuroprotective effects in a cuprizone-induced demyelination model [49]. Cdc42 itself has polar effects that regulate OL biology, especially those related to myelin sheath composition. It has been shown by ablation of Cdc42 and Rac1 that both exhibit stage specificity in myelin formation and synergistically regulate this critical stage [19]. Of course, the activation of Rho-family proteins requires a GEF to catalyze the exchange of GTP for bound GDP [50]. GEFs exert intense guanine nucleotide exchange activity on Cdc42 and Rac1 and promote their activation, thus inducing cellular morphological changes, protein backbone reorganization, and other events [10].

In this context, the present study aimed to investigate the effects of catalpol on myelin regeneration via the GEF-Cdc42/Rac1 signaling pathway. We hypothesized that catalpol could act in the same manner as triiodothyronine (T3) and activate the Integrin receptors on the cell membrane of OPCs, then activate GEFs in these cells; the activated GEFs would then convert GTP to GDP and transfer a phosphate off to Cdc42/Rac1 to phosphorylate it [20]. Phosphorylated Cdc42/Rac1 activates its downstream proteins PAK1 and MRCK*α*, which jointly maintain the differentiation capacity of OPCs. Therefore, catalpol may be involved in promoting OL differentiation by increasing the synergistic regulation of Cdc42 and Rac1. In our experiments, we performed double immunostaining for GEF and an OL marker (GST-pi) to show that catalpol treatment promoted the expression of GEF by OLs. Our data demonstrated that mice treated with catalpol had increased GEF expression and significantly increased number of OLs (GEF + GST-pi^+^). Furthermore, we investigated whether catalpol facilitates OL regeneration via the Cdc42/Rac1 signaling pathway. By double staining for Cdc42 and GST-pi, it was found that catalpol treatment promoted the expression of Cdc42 in OLs while increasing the number of Cdc42^+^GST-pi^+^ OLs, and Rac1 was consistent with the trend of the Cdc42 results. We found consistent results through western blotting and qRT-PCR experiments. The results of this study suggest that catalpol-mediated expression and activation of Rac1 and Cdc42 are critical for the outgrowth of OL processes. Along these lines, this study provides evidence that the mechanism through which catalpol treats EAE may consist of promoting the expression of GEF, which regulates the expression and activity of Cdc42 and its synergist Rac1, thereby promoting OL differentiation and myelin regeneration. Both Cdc42 and Rac1 are essential for the proper formation of myelin sheaths in the CNS and play key roles in guiding the development of OPCs. Catalpol promotes cytoskeletal expansion and branching through activation of Rac1 and Cdc42 expression in OLs, facilitating the differentiation process [18, 19].

There are limitations to our research. Considering that catalpol hardly penetrates the blood-brain barrier, its neural repair effect may be involved in the regulation of peripheral T cells, and the direct effect of catalpol on OPCs differentiation was not performed in vitro. Another limitation is that we did not perform toxicological markers testing in the present study.

## 5. Conclusions

Our study provides evidence that catalpol has potent anti-inflammatory activity, efficiently prevents demyelination, and promotes remyelination in the adult CNS. These results confirm that catalpol regulates OL regeneration and protects myelination via the GEF-Cdc42/Rac1 signaling pathway, alleviating the severity of EAE. In summary, catalpol is a promising therapeutic medication that effectively promotes nerve regeneration in a model of demyelinating disease.

## Data Availability

All data supporting the findings of this study are available from the corresponding author upon reasonable request.
